# A Dictionary of Practical Surgery

**Published:** 1887-01

**Authors:** 


### Reviews.

**A Dictionary of Practical Surgery, by various British Hospital Surgeons.** Edited by Christopher Heath, F. R. C. S., Holme Professor of Clinical Surgery in University College, London; Surgeon to University College Hospital; Member of the Council and Court of Examiners of the Royal College of Surgeons of England. In two volumes. Vol. I., Abdomen—Lymph—Scrotum; Vol. II., M—Z. Philadelphia: J. B. Lippincott Company. 1886.

The two volumes are bound together, thus making one quite large, yet not cumbersome, book. It is designed as a handbook of reference for the busy practitioner, and this purpose it subserves right well. Over one hundred eminent English medical men have contributed to this work articles of greater or less length. Such names as Paget, Thompson, Swain, Stokes Prestley, Smith, Poore, Page, Meredith, Liveing, Lawson, Langton, Gowers, Duckworth, Browne, Carter, Barlow, Anderson, and many others equally renowned, appear as contributors. "The subjects are treated of, so far as practicable, in the following order: 1. Cause; 2. Pathology; 3. Symptoms and Diagnosis; 4. Treatment; 5. Prognosis. Each writer has signed his articles, and is responsible for the statements contained in them; but the editor has, of course, exercised a general supervision, and has endeavored to prevent the promulgation of crude theories, or the inculcation of doubtful practices. At the same time, he has not attempted to reconcile the views of surgeons who may happen to differ on points of practice, nor does he endorse every statement put forth by the various writers. The aim of the editor has been to produce a compendium of the practice of British Surgery of the present day." At the end of the second volume, there will be found a general index. It is most complete, and greatly adds to the usefulness of the work. It will doubtless have a large sale, and take the place of the large encyclopædias, which have had their day.

